# A novel C-terminal degron identified in bacterial aldehyde decarbonylases using directed evolution

**DOI:** 10.1186/s13068-020-01753-5

**Published:** 2020-06-29

**Authors:** Yilan Liu, Jinjin Chen, Anna N. Khusnutdinova, Kevin Correia, Patrick Diep, Khorcheska A. Batyrova, Kayla Nemr, Robert Flick, Peter Stogios, Alexander F. Yakunin, Radhakrishnan Mahadevan

**Affiliations:** 1 Department of Chemical Engineering and Applied Chemistry, University of Toronto, 200 College Street, Toronto, ON M5S 3E5 Canada; 2 Centre for Environmental Biotechnology, School of Natural Sciences, Bangor University, Bangor, LL57 2UW UK; 3 Institute of Biomedical Engineering, University of Toronto, 200 College Street, Toronto, ON M5S 3E5 Canada

**Keywords:** Alkane, Aldehyde decarbonylase, Directed evolution, Degradation tag, Protease

## Abstract

**Background:**

Aldehyde decarbonylases (ADs), which convert acyl aldehydes into alkanes, supply promising solution for producing alkanes from renewable feedstock. However the instability of ADs impedes their further application. Therefore, the current study aimed to investigate the degradation mechanism of ADs and engineer it towards high stability.

**Results:**

Here, we describe the discovery of a degradation tag (degron) in the AD from marine cyanobacterium *Prochlorococcus marinus* using error-prone PCR-based directed evolution system. Bioinformatic analysis revealed that this C-terminal degron is common in bacterial ADs and identified a conserved C-terminal motif, RMSAYGLAAA, representing the AD degron (ADcon). Furthermore, we demonstrated that the ATP-dependent proteases ClpAP and Lon are involved in the degradation of AD-tagged proteins in *E. coli*, thereby limiting alkane production. Deletion or modification of the degron motif increased alkane production in vivo.

**Conclusion:**

This work revealed the presence of a novel degron in bacterial ADs responsible for its instability. The in vivo experiments proved eliminating or modifying the degron could stabilize AD, thereby producing higher titers of alkanes.

## Background

**Fig. 1:**
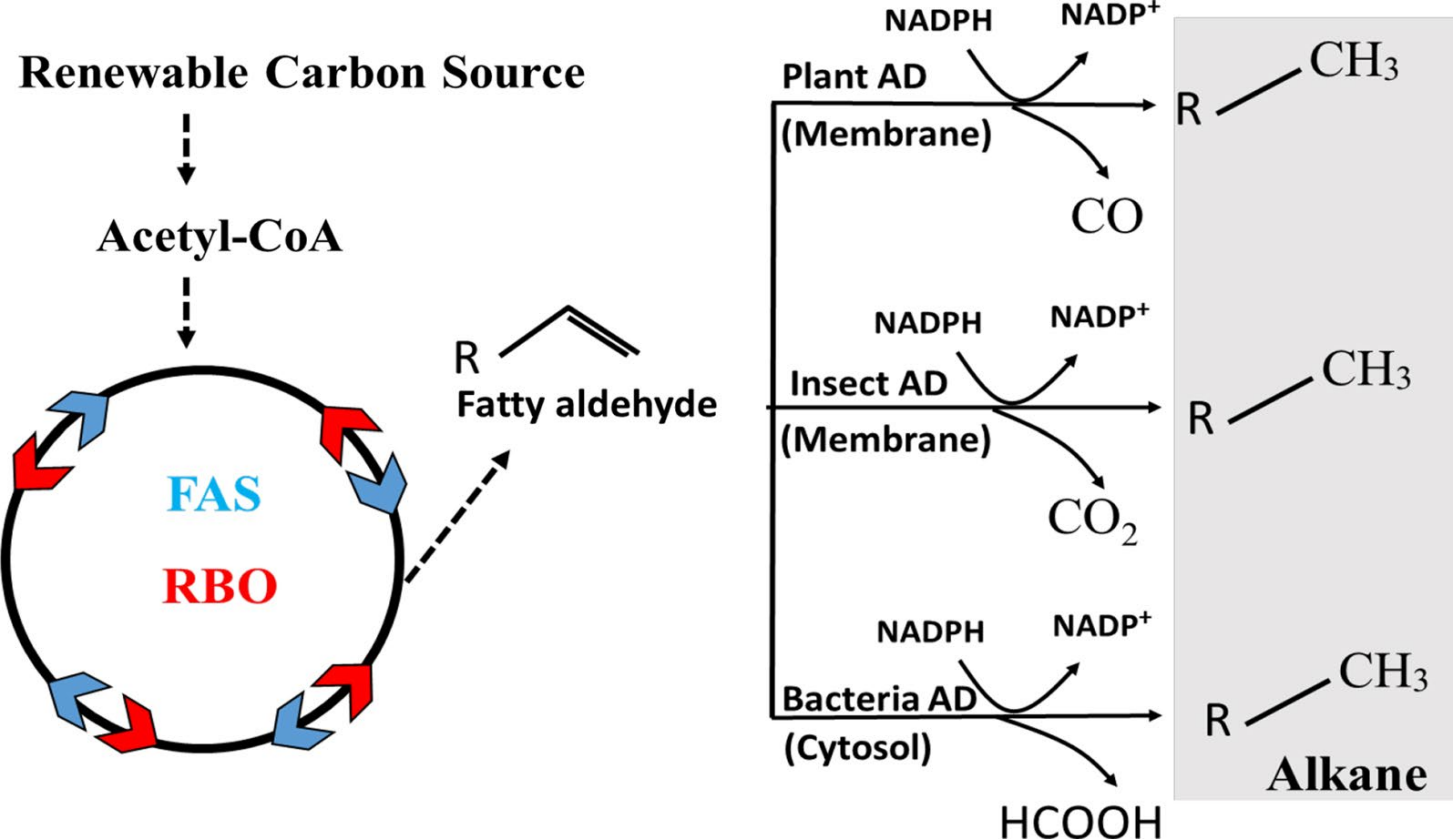
Metabolic strategies for alkane production. There are three distinct AD groups catalyzing the formation of alkane via different mechanisms. Both plant and insect ADs are membrane proteins, whereas bacterial ADs are localized in cytosol. *AD* aldehyde decarbonylase, *FAS* fatty acid synthesis, *RBO* reverse beta-oxidation

Rising energy consumption and the finite supply of fossil fuels are global challenges that demand sustainable alternative strategies [1–3]. Alkanes, the major components of conventional fuels [4], are mainly derived from non-renewable resources and can be naturally synthesized in cyanobacteria, green algae, insects and plants. Since Schirmer et al. (2010) identified two key enzymes responsible for alkane/ene biosynthesis in cyanobacteria [5], significant progress has been made in producing alkanes in microbial cell factories via fatty acid synthases (FAS) and reverse beta-oxidation (RBO) pathways and in vitro enzymatic electrosynthesis assisted by AD (Fig. 1) [6–13]. To our knowledge, the highest reported value for long chain alkane production is 2.54 g/L (C13-C17) in engineered *Escherichia coli* [7], but this is still insufficient for industrial scale-up. It has been reported that the low efficiency of AD is the bottleneck of alkane production [4, 14, 15], which warrants further investigation into how ADs behave in vivo.

Three distinct groups of ADs have been discovered (Fig. 1): AD in higher plants and green algae, that converts the aldehyde carbon to carbon monoxide [16–18]; AD in insects, a cytochrome P450 enzyme, which oxidizes the aldehyde carbon to carbon dioxide using the NADPH and molecular O_2_ [19, 20]; AD in bacteria, that converts the aldehyde carbon to formate [5, 21, 22]. Among these, both plant and insect ADs are membrane proteins, complicating their characterization in vitro. In contrast, bacterial ADs are small soluble proteins, which allows them to be studied in vitro more readily, leading to their wider use in metabolic engineering [11, 15, 23–26]. Although new bacterial AD is constantly being characterized and engineered for alkane production [11, 22, 23, 27, 28], our experiments showed that protein accumulation of bacterial ADs is limited in vivo. It was also reported that there is no significant accumulation of most bacterial AD in *E. coli* during incubation [15], and even a decrease in AD abundance after 10 h of incubation [28]. The low levels of AD might be due to fast in vivo degradation, which can limit the application of AD for alkane biosynthesis. This prompted us to attempt to improve the performance of bacterial ADs.

Directed evolution has proven to be a powerful strategy for improving enzyme properties [29–32].Therefore, we built up a directed evolution system to evolve bacterial ADs. Since high concentrations of aldehydes are toxic to *E. coli* cells, they can be used to establish a selection pressure to select cells containing AD variants with increased performance. In this study, error-prone PCR and hexanal were used in a directed evolution system and resulted in the discovery of C-terminal degron of AD from *Prochlorococcus marinus*. Since degrons are normally used to regulate protein expression and activity in cells [33, 34], we use bioinformatic and experimental approaches to better understand bacterial AD regulation and found that this C-terminal degron is conserved in the family of bacterial ADs. Knock-out strains were constructed and established that AD degradation occurs through protein recognition by ATP-dependent proteases ClpAP and Lon. Besides, our work proves that deletion or modification of the AD degron represents a novel strategy for developing efficient microbial cell factories for alkanes.

## Results

### Discovery of C-terminal degron via directed evolution

**Fig. 2:**
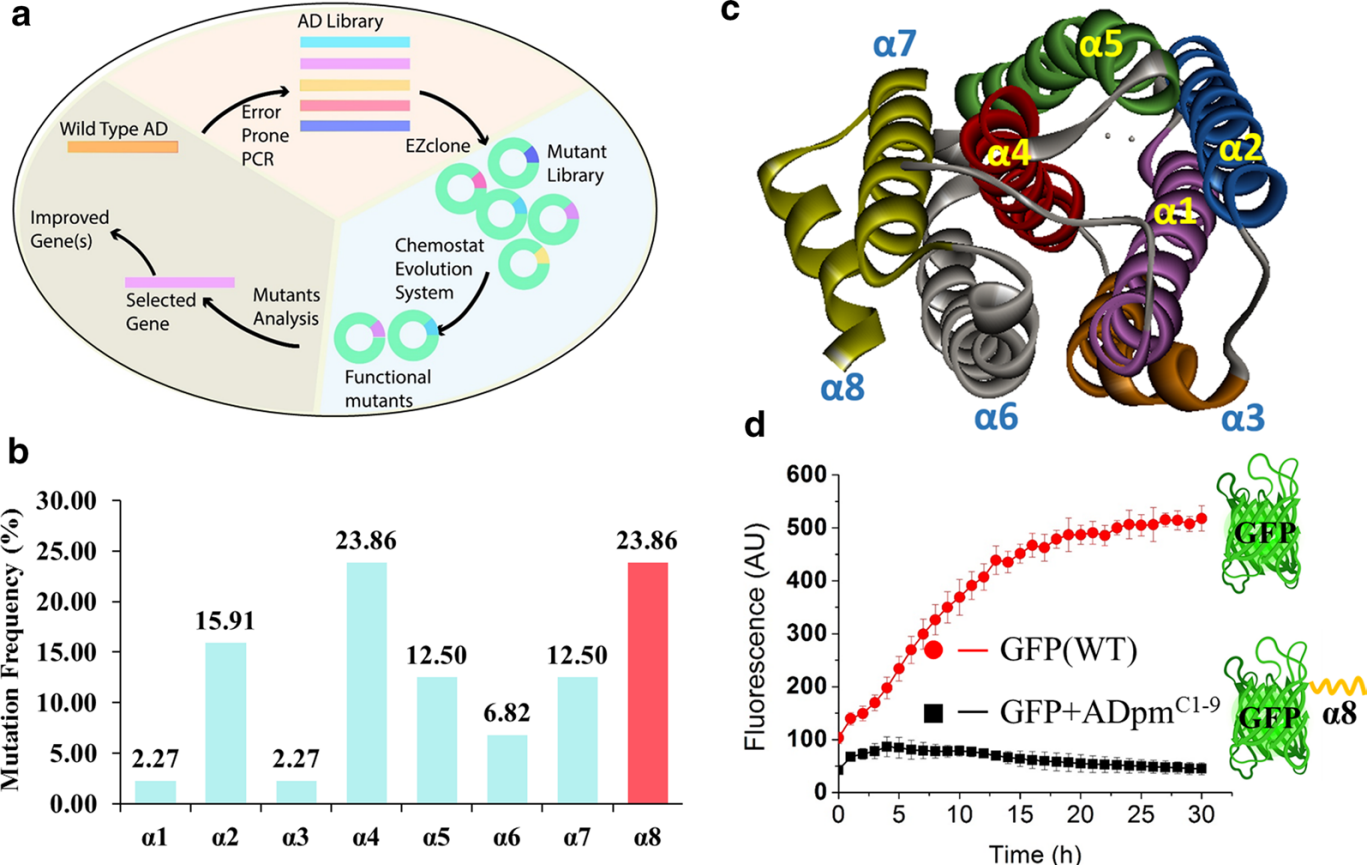
Discovery of a degron in ADpm via directed evolution. **a** Schematic of directed evolution system designed for AD evolution. **b** Distribution analysis of the mutations across the α-helices of ADpm. **c** Crystal structure of ADpm (PDBID: 2OC5). There are 8 α-helices in AD structure. α-helix 1, 2, 4 and 5 form the catalytic domain. **d** GFP degradation test with and without the ADpm^C1−9^ sequence. Fluorescence values were normalized to the number of cells by dividing by the OD_600_. Data show the mean value of five replicates

For directed evolution of ADs, the mutant variants of *Prochlorococcus marinus* AD (ADpm, Uniprot ID Q7V6D4) were generated using error-prone PCR, and *E. coli* cells were transferred to a chemostat cultivation system containing 2 g/L hexanal which was found to inhibit the growth of *E. coli* cells without recombinant AD. We hypothesized that this system would select for AD variants with increased rates of hexanal decarbonylation to pentane, which is less toxic (Fig. 2a).

After 7 days of chemostat cultivation, 33 mutants were sequenced and the distribution of mutations in the AD coding sequence was analyzed. The highest mutation frequency was observed in the α4 and α8 helices (Fig. 2b), and the α8-truncated mutants, due to the nonsense mutations, were found to have the highest activity based on the amount of NADPH consumed (Additional file 1: Figure S1). According to the crystal structure of ADpm, eight α-helices form the intact structure, among which α1, 2, 4 and 5 form the catalytic domain (Fig. 2c). Mutations in these domains might benefit the activity of AD. Since α8 helix is far from the catalytic domain, we hypothesized that α8-truncated mutants might be filtered out due to a novel mechanism other than enhanced activity.

This was further probed in vitro, by expressing ADpm without the C-terminal helix, RMAAAALVS (ADpm^C1−9^), herein termed ADpm-9. Enzymatic assays with purified ADpm-9 proteins revealed a decrease in specific activity and thermostability, whereas size-exclusion chromatography showed no change in the oligomeric state compared to ADpm (Additional file 1: Figure S2). The results lead us to hypothesize that *E. coli* expressing ADpm-9 exhibited improved alkane production because the biostability of the enzyme was improved, thus increasing its abundance. As ADpm^C1−9^ shows rich of L, A, V and S as *E. coli* degron motif 1 (Additional file 1: Figure S3) [35], so ADpm^C1−9^ was speculated to be a degron that shortens the half-life of ADpm. To test this hypothesis, the ADpm^C1−9^ was fused to green fluorescent protein (GFP) to monitor degradation over time in vivo. The fluorescence increased slightly in the beginning four hours for *E. coli* expressing ADpm^C1−9^-tagged GFP, but decreased during the remainder of the incubation. In contrast, the fluorescence of untagged GFP increased till 19 h after inoculation and remained stable thereafter (Fig. 2d). Overall, ADpm^C1−9^ reduced the GFP fluorescence to 8.6% of the untagged GFP after 30 h of cultivation, which supports the hypothesis that the short C-terminal region of ADpm functions as a degron reducing protein half-life in vivo.

### Identification of the minimal AD degron sequence

**Fig. 3:**
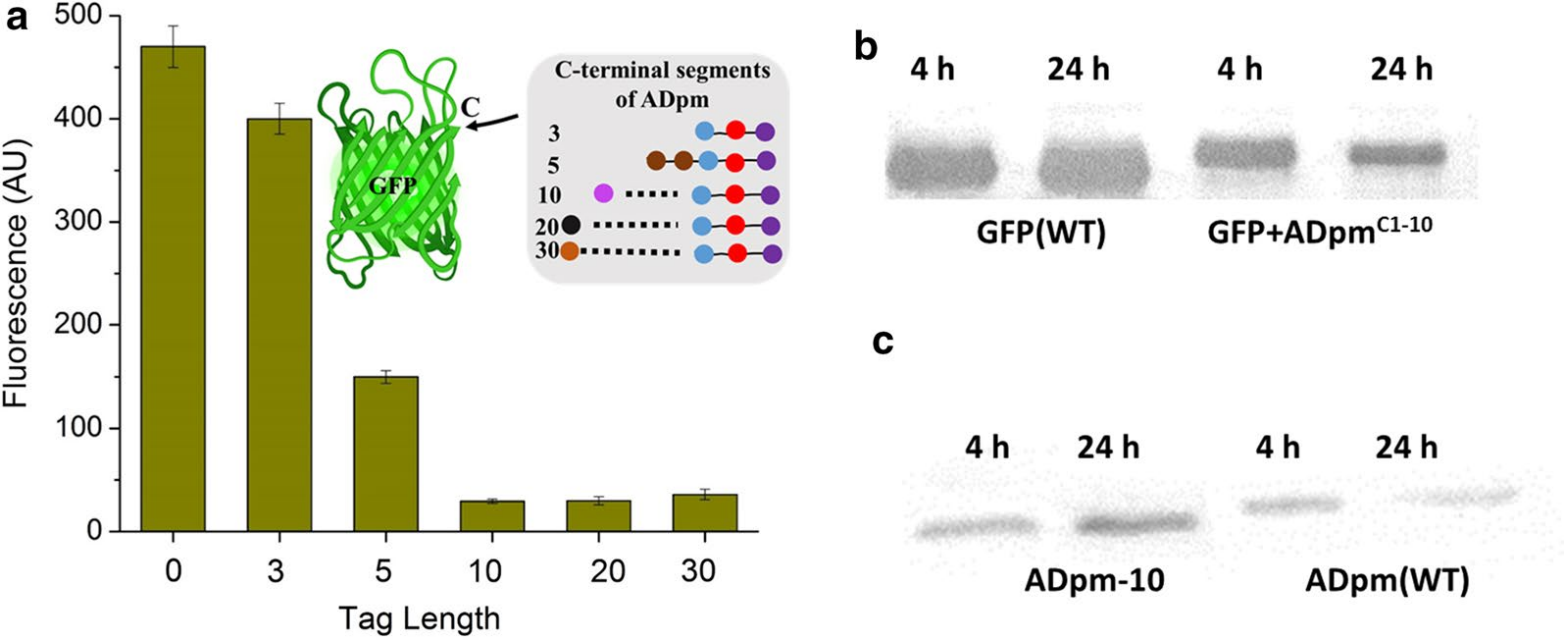
Degradation effect of C-terminal region of AD. **a** Fluorescence of GFP fused with different C-terminal sections of AD at 24 h after inoculation. **b** Western blot for GFP with and without 10 amino acids from the C terminus of AD. **c** Western blot for AD with and without elimination of 10 C-terminal sequence

To more precisely determine which part of the C-terminal region of ADpm is necessary for its degradation, GFP was tagged with different C-terminal segments of ADpm (3, 5, 10, 20, and 30 amino acids). Each tagged GFP was controlled by a constitutive synthetic promoter pJ23119. Compared to the untagged GFP, degradation was observed in all the tagged GFP strains (Fig. 3a). The degradation of GFP increased as the degron length increased from 3 (GFP + ADpm^C1−3^) to 10 (GFP + ADpm^C1−10^), but no further enhancement of degradation was observed beyond 10 residues (Fig. 3a). These results indicate that the short C-terminal region of ADpm (10 amino acids) is the minimal AD degron sequence.

Western blot was used to analyze the protein degradation of GFP and ADpm triggered by the C-terminal degron. Fluorescence assays revealed a 90% decrease in fluorescence of GFP + ADpm^C1−10^ compared to the untagged GFP, which is in line with approximately 90% reduction in the GFP protein level for the GFP + ADpm^C1−10^ construct (Fig. 3a,b). These results demonstrate fluorescence from the GFP protein is directly proportional to the abundance of the GFP protein. The protein concentration of untagged GFP increased with more incubation time, while the fluorescence of GFP + ADpm^C1−10^ decreased. Similarly, the protein concentration of wild-type ADpm decreased with more incubation time while protein accumulation was observed in ADpm-10 (with 10 C-terminal amino acids missing) (Fig. 3c). These results indicate that a short C-terminal region of ADpm, ADpm^C1−10^, represents a degron whose presence leads to proteolytic degradation in vivo.

### Recognition of the C-terminal degron in bacterial ADs via bioinformatic analyses

**Fig. 4:**
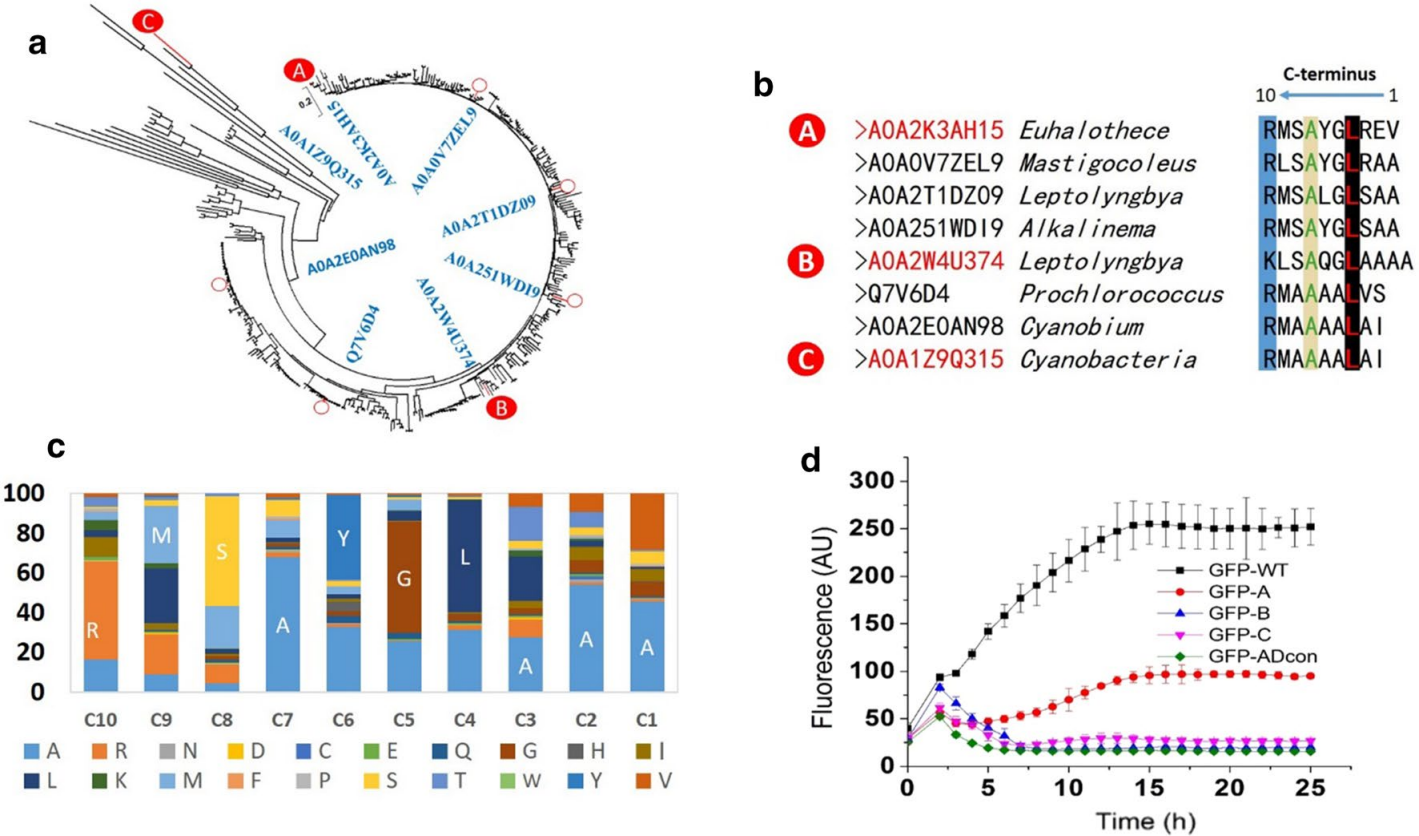
Analysis of C-terminal sequences of AD homologs in bacteria. **a** Phylogenetic reconstruction of AD sequences. The phylogenetic tree was generated using MEGA 5.10’s NJ method with 100 bootstrap replications. **b** Multiple sequence alignment of the C-terminal regions of eight AD sequences, with three representatives (labeled **a, b, c**) used for subsequent investigation. **c** Statistical analysis of C-terminal sequence of 371 AD homologs. **d** GFP degradation test of three representative C-terminal peptides and the conserved degron sequence. GFP-A, GFP-B, GFP-C represent GFP fused with three C-terminal regions from (**b**). GFP-ADcon represents GFP fused with conserved sequence from (**c**). Fluorescence values were normalized to the number of cells by dividing by the OD_600_. The data shown are from the mean of three biological replicates

To expand our understanding of biostability of the bacterial ADs, over 600 protein sequences of bacterial AD homologues from the UniProtKB database were analyzed using multiple sequence alignments. This dataset was reduced to 371 sequences by removing redundant sequences. A phylogenetic tree was generated using MEGA 5.10’s NJ method with 100 bootstrap replications (Fig. 4a). For further analysis, eight representative sequences from different branches were selected from the phylogenetic tree. A high degree of conservation was observed in C-terminal residues of the selected candidates (Fig. 4b), including basic amino acids (Arg or Lys) at the 10th position and nonpolar Ala and Leu at the 7th and 4th positions, respectively. Ala–Ala dipeptides were also observed to be a common feature in the C terminus of ADs (Fig. 4b). These results suggest that the C-terminal degron detected in ADpm appears to be a conserved motif in the family of bacterial ADs. A statistical analysis was conducted for the last 10 amino acids of the C-terminal sequence from ADs in the reduced dataset. It was revealed that half of the positions have an amino acid frequency larger than 50% (Additional file 1: Table S1). The most conserved position is Ala at the 7th position with a frequency of 67.92% among the bacterial AD homologs (Fig. 4c). From this, RMSAYGLAAA appears to be a consensus sequence for a degron conserved in bacterial ADs, herein termed ADcon.

To test this hypothesis and evaluate the functionality of ADcon, the C-terminal sequences of three ADs from major branches of the phylogenetic tree, as well as the ADcon, were fused to GFP to test whether they would trigger protein degradation. All four C-terminal sequences caused marked GFP degradation (Fig. 4d), which proved that the C-termini of bacterial AD homologs serve as degrons. Furthermore, it was observed that lack of some of the conserved residues affected the degron efficacy. For example, the degron from *Euhalothece* (RMSAYGLREV), which lacked the Ala–Ala dipeptides, only caused 62% of GFP degradation after 25 h incubation compared to the ADcon which caused 94% GFP degradation. Besides, it was reported that AD from *Gloeobacter violaceus* PCC 7421 (7421ADO) shows higher protein level in *E. coli* than AD from cyanobacterial strains [15]. Based on sequence alignment, we observed significant amino acid difference in the degron region of 7421ADO, suggesting that 7421ADO is less susceptible to proteolytic degradation explaining a higher protein level. These findings strongly support our hypothesis that the C-termini of bacterial ADs are a conserved degron.

### Investigation of molecular mechanisms of AD degron-dependent protein degradation in *E. coli*

**Fig. 5:**
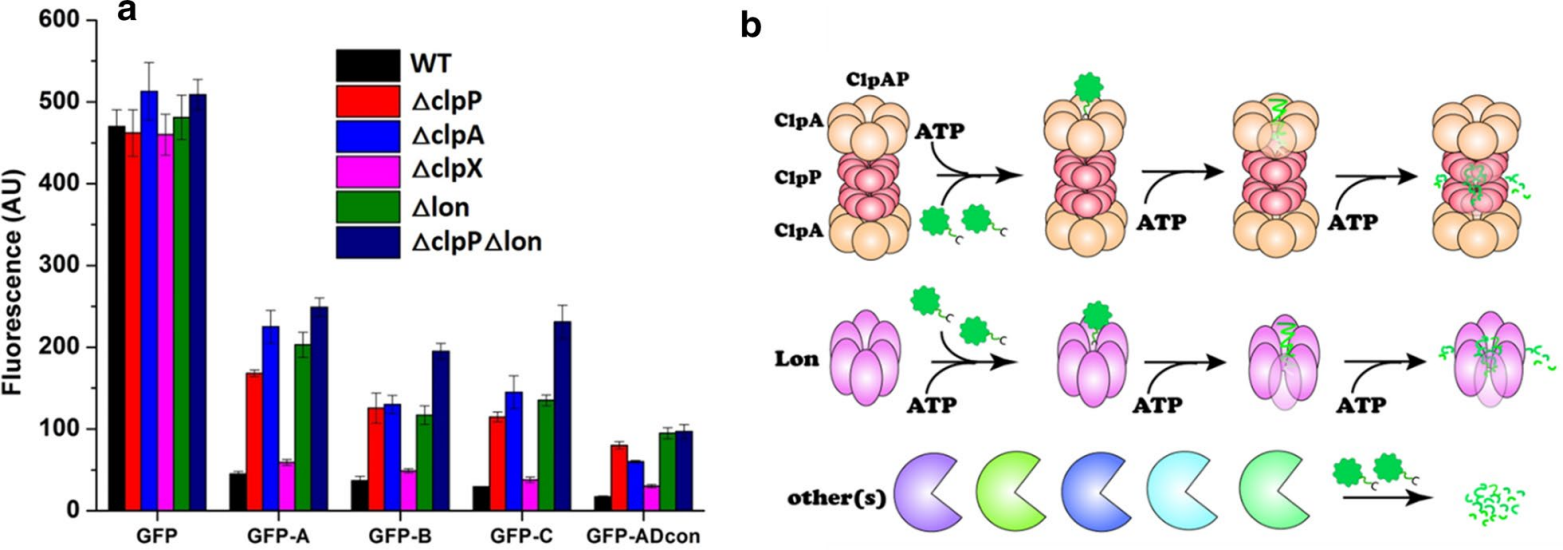
Investigation of degradation mechanism of AD degron. **a** Effects of protease deletion on GFP degradation with data normalized by OD_600_. **b** Diagram illustrating the recognition and degradation process of AD degron-tagged protein. GFP-A, GFP-B, GFP-C and GFP-ADcon refer to Fig. 4d. AD degron can be recognized by the ClpA and Lon protease complexes. ClpA-bound GFP will be unfolded and translocated into ClpP for subsequent degradation. Lon-bound GFP will be unfolded and degraded simultaneously in itself. Fluorescence values were normalized to the number of cells by dividing by the OD_600_. The data shown are from the mean of three biological replicates

To pinpoint the mechanism of protein degradation triggered by the AD degron, ADcon was compared to previously reported C-terminal degrons in *E. coli* [35]. We found that ADcon shares sequence similarity to the ssrA degron (CAANDENYALAA) from *E. coli*, which is degraded by the ClpAP and ClpXP protease complexes [36]. To test the hypothesis that the degradation mechanism of AD degron is like ssrA’s, degron-tagged GFP was transformed into constructed ∆ClpA, ∆ClpX, and ∆ClpP strains. In all four degron-tagged GFP strains, fluorescence recovery was observed in both ∆ClpA and ∆ClpP strains, but not significant in the ∆ClpX strain, compared to WT *E. coli* (Fig. 5a). This suggests that AD degron triggers protein degradation using the ClpAP protease complex where ClpA is responsible for recognizing the signal peptide, unfolding the protein, and translocating it to ClpP for proteolysis (Fig. 5b).

However, GFP degradation was still observed in the ∆ClpP strain compared with the untagged GFP (Fig. 5a). Previous studies found that a single degron can be recognized by multiple protease complexes. This includes the Umu degron that is recognized by the Lon protease complex and ClpXP, as well as the ssrA degron that can be recognized by both ClpXP and ClpAP [37–39]. Lon protease was suspected to degrade AD degron-tagged GFP since it is known to be an efficient protease for non-native protein degradation [40]. To test whether Lon protease can recognize AD degron, we transformed the AD degron-tagged GFPs into a constructed ∆Lon *E. coli* strain. The fluorescence was partially recovered in all tagged candidates when expressed in the *E. coli* ∆Lon strain. Nonetheless, GFP degradation still occurred in all degron-tagged GFP samples even in the ∆ClpP∆Lon strain (Fig. 5a). These results suggest that there are other proteases that can recognize AD degron in *E. coli*. The protease system in bacteria is complicated and vital for all biological pathways [41], which was supported by our observation that the physical appearance of the cell cultures bearing Lon and ClpP knock-outs had a stickier consistency compared to wild-type cells which are normally pasty. Hence, the need to maintain cell viability prevents the deletion of all proteases responsible for AD degron recognition in order to improve the biostability of AD, leaving only the option of managing the impact of the degradation tag.

### Effects of AD degron engineering on alkane production

**Fig. 6:**
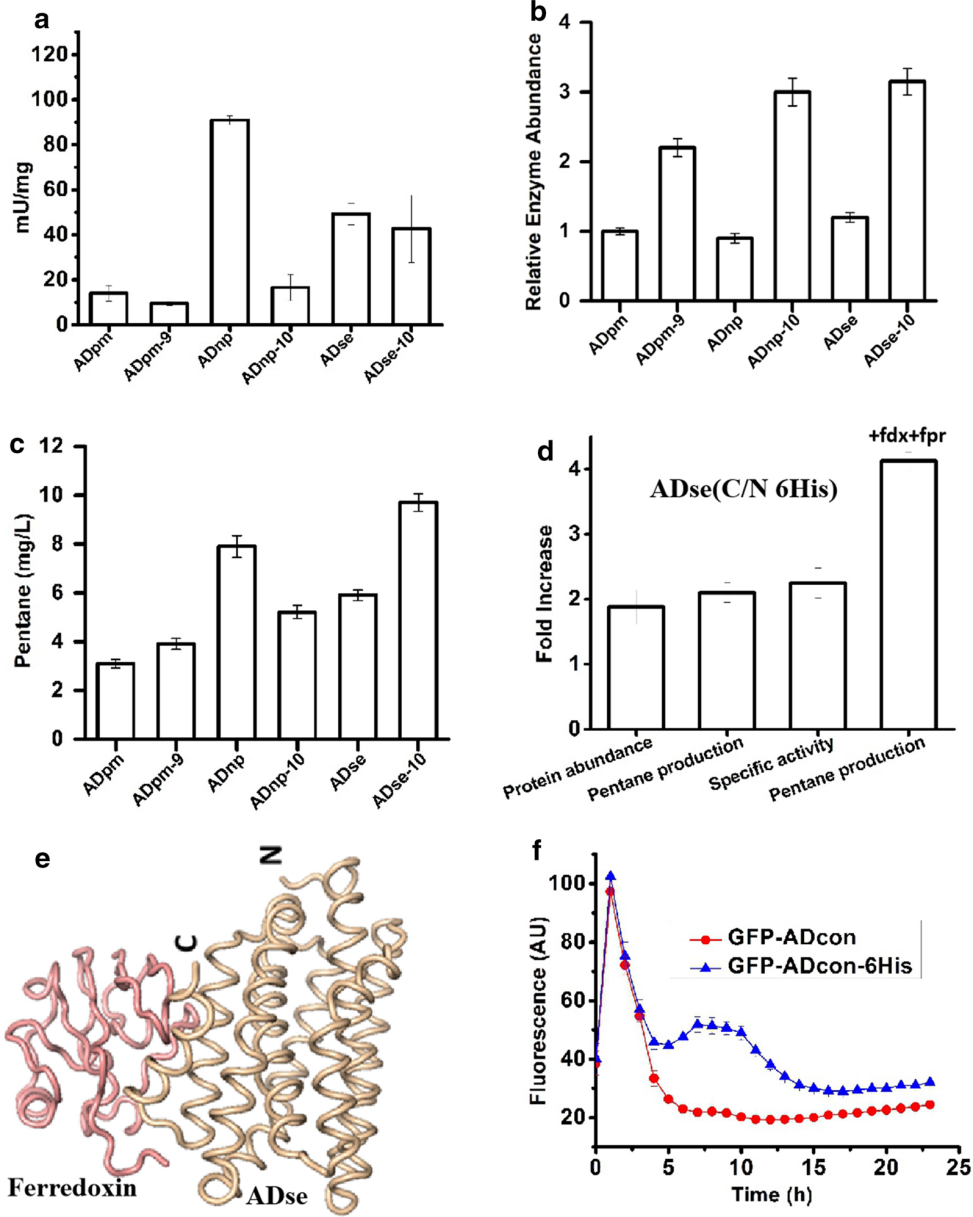
Investigation of degron modification. Representative ADs from *P. marinus* (ADpm), *N. punctiforme* (ADnp) and *S. elongatus* (ADse). **a-c** Comparison of specific activities, enzyme abundance and pentane production in three representative ADs with and without degron. Enzyme abundance was normalized to ADpm based on peak area. **d** Comparison of enzyme abundance and pentane production in ADse C-terminal His-tag and N-terminal His-tag. **e** Modeling of protein–protein interaction between ADse and ferredoxin from *E. coli*. *E. coli* 2Fe2S ferredoxin (Uniprot ID P0A9R4) (colored pink) and ADse decarbonylase (colored wheat) interaction model predicted with GRAMMX protein–protein docking server. The C- and N-terminal ends of ADse are designated. **f** C-terminal His-tag effect on degron sealing. The data shows the mean of three replicates, and all error bars show the S.D

The three most commonly used ADs from *Prochlorococcus marinus* (ADpm, Uniprot ID –Q7V6D4), *Nostoc punctiforme* (ADnp, Uniprot ID B2J1M1) and *Synechococcus elongatus* (ADse, Uniprot ID Q54764) were selected to investigate the effects of degrons modification on alkane production. First, degron parts were removed from all three ADs to create ADpm-9, ADnp-10, and ADse-10. Initial enzymatic screening revealed the specific activities of degron-free versions of ADpm, ADnp and ADse decreased from 14.9 to 9.6 (1.55-fold decrease), 90.9 to 16.6 (5.47-fold decrease), and 49.1 to 42.7 (1.14-fold decrease) mU/mg, respectively (Fig. 6a). In contrast, the relative enzyme abundance of degron-free versions increased 2.2-, 2.65- and 3.3-fold in cells harboring ADpm, ADnp, ADse, respectively (Fig. 6b). Consequently, pentane production increased from 3.1 to 3.9 mg/L with ADpm and from 5.9 to 9.7 mg/L with ADse in *E. coli* (Fig. 5c). We attribute this improvement in pentane production to the increase in AD protein abundance, which not only compensates for the reduction in enzyme activity, but also increases overall pentane accumulation. The pentane production decreased from 7.9 to 5.2 mg/L in *E. coli* harboring ADnp-10 compared with ADnp (Fig. 6c), because its 2.65-fold increase in enzyme abundance could not compensate the 5.47-fold decrease in activity. Our modeling results suggest that the elimination of the C-terminal sequence has a negative effect on the substrate binding in all three candidates (Additional file 1: Figure S4), which is consistent with the notion that residues far from the active site and the substrate binding site still contribute to the enzyme activity of AD [15].

Since deleting protease complexes negatively impacts cell viability and deleting the entire AD degron decreases enzyme activity, an alternative method to increase the half-life of AD is needed. We hypothesized that addition of amino acids after the degron would protect the AD from proteolytic degradation. This was tested by adding a 6xHis-tag after the native degron in wild-type ADse, which previously produced the most pentane (Fig. 6c). As shown in Fig. 6d, ADse with a 6xHis-tag shows 1.9-fold and 2.1-fold increases in protein abundance and pentane production, respectively, compared to wild-type ADse. Surprisingly, the 6xHisTag also improved the specific activity, possibly because the addition of His-tag affects the conformation of the C-terminal helix and somehow benefit the activity of ADse. Our docking results also provide some support for this hypothesis. One of the two highest scored interacting models shows that ferredoxin can interact with the ADse C-terminal helix (Fig. 6e), indicating that the C-terminal helix modification might improve the recruitment of ferredoxin (Fig. 6d). Turning to pentane production, the highest titer comes with overexpression of fdx and fpr (Fig. 6d), suggesting that ferredoxin (fdx) and ferredoxin reductase (fpr) are necessary for electron transfer to AD and for high enzyme activity. The protection principle was extended to GFP + ADcon in vivo to test the limit of the protective effect conferred by a 6xHis-tag. Figure 6e shows that the 6xHis-tag protects GFP + ADcon from protein degradation during the cultivation (Fig. 6f). However, in comparison to wild-type GFP, the presence of 6xHis-tag did not totally prevent the degron recognition by protease (Additional file 1: Figure S5). Overall, these results demonstrate that manipulating the AD degron can improve enzyme activity and alkane production.

## Discussion

Given the importance of alkanes for liquid transportation fuels, engineers have sought to find sustainable ways to produce alkane using the bacterial AD-based pathway [5, 7, 8]. AD was previously diagnosed as the bottleneck in the pathway [4]. It was reported that AD expressed in *E. coli* had low abundance and degradation problems [28]. However, the mechanism remained elusive, yet crucial for microbial fermentation application. In this study, we discovered a C-terminal degron in *P. marinus* AD via a directed evolution system, which has been successfully employed to improve activity of enzymes through the enhancement of solubility, thermostability, substrate affinity, and catalytic turnover [32]. For the first time, the directed evolution system was employed to discover a degron. Since degrons are ubiquitous in N-terminal, C-terminal, and internal regions of various proteins [35, 42, 43], our study marks new avenues to use directed evolution to explore protein degrons, and to correct them for improved enzyme abundance and potentially enzyme activity.

More importantly, a C-terminal degron (AD-con) was found to be conserved in the family of bacterial AD, which was surprising because degrons normally exist in stress proteins expressed in response to environmental changes [35]. This might be the result of its promiscuous substrate specificity that would convert metabolically vital aldehyde intermediates into non-recyclable alkane/enes in bacteria, ultimately resulting in a loss of carbon. Our product analysis showed the presence of various volatile compounds in the culture headspace (Additional file 1:Figure S6). Therefore, we suggest that the existence of degron can protect microorganisms from losing carbon via volatile products. Growth curves of *E. coli* harboring ADse and ADse-10 in M9 medium containing different glucose concentrations were tested and showed a decrease in the growth rate in ADse-10 strain under all conditions (Additional file 1: Figure S7), which supports the notion that the AD degron provides a fitness advantage during evolution. In addition, it was reported that the expression of bacterial genes involved in the assimilation of alkanes is usually tightly regulated [44], which also suggests that there are regulatory processes present in the alkane metabolic pathway.

Our bioinformatics and experimental investigation strongly suggest that virtually all bacterial ADs possess a C-terminal degron, and its elimination can increase alkane production in vivo, albeit negatively affecting the catalytic efficiency. We also showed that one of the approaches for mitigating the proteolytic degradation of the degron is adding extra amino acids after the native degron to protect it from protease recognition. This modification could confer moderate improvements in AD half-life and activity, ultimately improving alkane production. However, additional methods for managing the trade-off between activity and stability are needed in order to further enhance alkane production. Additionally, any increase in activity and stability also must address the specificity of AD toward the desired aldehydes relative to other aldehyde intermediates in central metabolism. In this study, we have taken the first step towards addressing this trade-off through the comprehensive characterization of the role of the C-terminal degron in AD and its impact on enzyme stability and activity. We anticipate that this work will contribute to transitioning AD-based biosynthesis of alkanes to industrial scales.

## Conclusion

For the first time, a degradation tag (degron) was discovered in the AD from marine cyanobacterium *Prochlorococcus marinus* using error-prone PCR-based directed evolution system. Bioinformatic analyses were carried out and revealed that this C-terminal degron is common in the family of bacterial ADs and identified a conserved C-terminal motif, RMSAYGLAAA, representing the AD degron (ADcon). Furthermore, we demonstrated that the ATP-dependent proteases ClpAP and Lon are involved in the degradation of AD-tagged proteins in *E. coli*, thereby limiting alkane production. Deletion or modification of the degron motif increased alkane production in vivo. Thus, our work revealed the presence of a novel degron in bacterial ADs and paves way for its engineering for microbial production of sustainable fuels.

## Methods

### General molecular methods

*Escherichia coli* K-12 strains MG1655 was used for directed evolution and degradation mechanism investigation. RARE [4] strain was used for in vivo fermentation. Random mutagenesis of AD by error-prone PCR was performed with the GeneMorphII Random Mutagenesis Kit (Agilent Technologies) with initial template amount 100 ng, PCR cycle number 25 to achieve low mutation frequency (0–4.5%). ADs were cloned into pZa23MCS without codon optimization. Plasmids and DNA were purified using Monarch kits (NEB) according the manufacturer’s instructions. Restriction digests were carried out using standard protocols of NEB restriction endonucleases. For ligation of DNA fragments, T4 DNA ligase (Life Technologies) was used according to manufacturer’s instructions. Gene deletions were performed by red-mediated recombination [45]. The I-TASSER Suite was employed to generate structural model of ADs minus degrons [46]. Autodock Vina was used for molecular docking [47]. Protein stability was predicted by SCooP [48]. GRAMMX server was used for protein–protein interaction prediction [49].

### Culture conditions

For fluorescence or fermentation, cells were culture in LB (Luria–Bertani) medium. For in vivo pentane synthesis, seed cultures were grown in LB medium at 30 °C overnight on a rotary shaker at 250 rpm, and were used to inoculate, at an inoculation volume of 1%, 1 mL LB in 15-mL tube for aerobic growth. Cultures were induced with 1 mM IPTG at OD_600_ of 0.6. Cells were transferred to anaerobic fermentation 4 h after IPTG induction and re-inoculated into 1 mL LB culture with 10 g/L glycerol, 1 mM IPTG and 1 g/L hexanal in sealed bottle for anaerobic fermentation 12 h. The headspace was used for pentane quantification and the culture was used for protein-level analysis. In general experiments were performed in triplicate, and the data are given as the averages and SDs. For protein purification, starter cultures were grown in LB medium at 37 °C overnight on a rotary shaker at 250 rpm, and were used to inoculate, at an inoculation volume of 2%, 1 L TB (Terrific Broth) medium. Cultures were grown aerobically at 37 °C until OD_600_ between 0.8 and 1, induced with 0.4 mM IPTG and left at 37 °C for 5 h. Biomass was collected by spinning at 3000 g, and frozen at − 20 °C for purification.

### Western blots

The protein abundance analysis, Western blot was carried out. Whole cell lysis was separated on a 18% SDS-PAGE gel. Sample amount was equaled based on culture OD. Proteins on gel were transferred to a nitrocellulose membrane and detected by a primary 6x-His-tag Antibody (ThermoFisher, USA) from mouse and goat anti-mouse conjugated to alkaline phosphatase or HRP as a secondary antibody (Bio-Rad). Protein with His-tag on the nitrocellulose membrane was detected by using Opti-4CN kit (Bio-Rad). The intensity of western blot bands was analyzed using ImageJ.

### GC–MS and GC-FID methods

For the analysis of short chain alkanes produced, the sample headspace was analyzed by EI GC–MS. GC–MS analyses were performed on a Varian 2100T, with the detector in EI and operated at 10 eV. An Agilent VF-5 ms column (30 m length, 0.25 mm inner diameter, 0.25 µm film thickness) was used at 50:1 split ratio. The ion source temperature was set to 200 °C. GC analyses were performed as follows. The oven temperature was maintained at 40 °C for 4 min. Temperature was increased at a rate of 50 °C/min up to 200 °C and maintained for 1 min, followed by an increase of 20 °C/min up to 270 °C and maintained for 3.3 min. And 0.5 mL of headspace was injected. The retention times and fragmentation patterns were compared with those obtained from pure standards. Pentane was quantitatively analyzed by GC-FID. GC-FID analysis was performed with a Hewlett–Packard 5890 series II gas chromatograph equipped with a flash heat split inlet; and 30 m long, 0.53 mm id capillary column (J&W Scientific). The GC program was as follows: initial temperature of 40 °C, hold for 4 min; then ramp to 190 °C at a rate of 40 °C per min and hold for 6 min.

### Enzyme kinetics

Whole cell lysis activity analysis was carried in 100 mM PBS buffer pH 7.5, 0.1 M KCl, supplied with 0.2 mM FeCl_2_, 8 mM hexanal and 2 uL of each cell lysis in 100 uL volume. Purified Enzyme assay was carried in 100 mM HEPES pH 7.5, 0.1 M NaCl, supplied with 2 mM NADH, 0.3 mM FeCl_2_, 0.1 mM PMS, 10 mM hexanal and 50 µg of each decarbonylase in 0.2 mL liquid volume, 1.8 mL N_2_ gas phase in gas tight vials. Reaction premix and enzymes were degassed and sparged with argon. Water for reaction mixture dilution was degassed, sparged with argon and oxygen leftovers were removed by titanium citrate. Reaction was started in N_2_ atmosphere glove box, vials were tightly capped and incubated on shaker at 200 rpm, 37 °C. Reaction was stopped by heating at 100 °C for 5 min. 200 µL of gas phase were analyzed using GC–MS protocol.

### Protein purification

*Escherichia coli* cell thawed biomass was diluted in binding buffer (50 mM HEPES, pH 7.5, 0.4 M NaCl, 5% glycerol, 5 mM imidazole) and sonicated on ice bath during 15 min following the 3-s ON (120-140 W) and 4-s OFF regime using Qsonica Q700 equipped with dual-horn probe. Cell lysate was spun down at 37,000 g for 30 min, 4 °C, and supernatant was applied to the Ni-agarose equilibrated with binding buffer. Resin was washed with 50 volumes of washing buffer (50 mM HEPES, pH 7.5, 0.4 M NaCl, 5% glycerol, 30 mM imidazole) and eluted with 3–6 mL of elution buffer (50 mM HEPES, pH 7.5, 0.4 M NaCl, 5% glycerol, 250 mM imidazole). Proteins were frozen in droplets using liquid N_2_ and stored at − 80 °C.

### Analytical size-exclusion chromatography

Analytical size-exclusion chromatography was performed on Superdex 200 16/60 GL column. The equilibration buffer contained 50 mM HEPES pH 7.5, 0.1 M NaCl. For oligomeric state testing 1-2 mg of protein was loaded to the column and eluted for 2 column volumes. Relative protein size was estimated based on BioRad chromatography protein standards run.

### Fluorescent assay with whole cells

The degradation of GFP was quantified using a fluorescent assay. For this purpose, cells were cultured in 24-well plates. The fluorescence was monitored by fluorescence in a microtiter plate reader (excitation wavelength, 488 nm; emission wavelength, 535 nm; Infinite M1000 PRO, Tecan Group AG) at 37 °C and agitation (6 mm amplitude, orbital). Fluorescence signal in the linear range of the reaction was normalized by the OD_600_ of the respective culture.

### Thermal shift assay

Protein thermostability (Tm, melting temperature) was determined using SYPRO Orange dye kit (ThermoFisher) on the CFX96 Touch Real-time PCR Detection System reading FRET channel on λ_ex_ 492 nm and λ_em_ 610 nm. Reaction volume was 20 µL, protein concentration around 0.1 mg/mL. In order to optimize the fluorescence signal-to-noise ratio, an assay optimization was performed.

## Supplementary information

**Additional file 1: Figure S1.** Whole cell lysis activity analysis of different mutants. M2 give the highest activity, which has a premature stop codon in the C-terminal region. *WT* wild type ADpm, M1–M9 different mutants. **Figure S2**. Comparison of ADpm and ADpm-9. **a** Specific activity of ADpm and ADpm-9. **b** Relative protein thermal stability estimated using SYPRO Orange dye. **c** Investigation of oligomeric state of ADpm and ADpm-9 using size exclusion chromatography. **Figure S3.** Comparison of C-terminal of ADpm and C-motif 1 [35] of protease trapped proteins. **Figure S4.** Modelling results of degron subtraction in three representative ADs from *P. marinus* (ADpm), *N. punctiforme* (ADnp) and *S. elongates* (ADse). **a** Thermostability and hexanal docking analysis of three ADs and their C-terminal truncations. (**b–d**) Examples of docking analysis in ADpm and ADpm-9. **b** ADpm, **c** ADpm-9, **d** Superimposed structures. Note, residues shown in red indicate the interact amino acids keep consistent in the truncated and wild-type ones. **Figure S5.** Effect of C-terminal His-tag on GFP degradation. C-terminal 6His-tag was added to GFP with conserved degron. **Figure S6.** Effects of AD degron elimination on fermentation. Headspace analysis of ADse (Red) and ADse-10 (Blue). U1–U7, unidentified peaks. **Figure S7.** Effects of AD degron elimination on cell growth. (**a–d**) Growth curves of ADse (black) and ADse-10 (red) in M9 medium containing different concentrations (0.5, 1, 2 and 10 g/L) of glucose. **Table S1.** Statistical analysis of amino acid usage frequency (%) at C-terminus in 371 bacterial ADs.

## Data Availability

All data are included in the manuscript.

## Abbreviations

AD: Aldehyde decarbonylase
fdx: Ferredoxin
fpr: Ferredoxin reductase
PMS: Phenazine methosulfate
clpA: ATP-dependent protease
clpP: Serine protease
clpX: ATP-dependent protease
lon: Lon protease
Umu: umuC and umuD DNA polymerase
ssrA: Transfer-messenger RNA
PMS: Phenazine methosulfate
