# Supplementary material for: A novel C-terminal degron identified in bacterial aldehyde decarbonylases using directed evolution
Source: Biotechnol Biofuels. 2020 Jun 29;13:114. doi: 10.1186/s13068-020-01753-5 (PMC7325246; doi:10.1186/s13068-020-01753-5)
Supplement: Supplementary file 1 — Additional file 1: Figure S1. Whole cell lysis activity analysis of different mutants. M2 give the highest activity, which has a premature stop codon in the C-terminal region. WT wild type ADpm, M1–M9 different mutants. Figure S2. Comparison of ADpm and ADpm-9. a Specific activity of ADpm and ADpm-9. b Relative protein thermal stability estimated using SYPRO Orange dye. c Investigation of oligomeric state of ADpm and ADpm-9 using size exclusion chromatography. Figure S3. Comparison of C-terminal of ADpm and C-motif 1 [35] of protease trapped proteins. Figure S4. Modelling results of degron subtraction in three representative ADs from P. marinus (ADpm), N. punctiforme (ADnp) and S. elongates (ADse). a Thermostability and hexanal docking analysis of three ADs and their C-terminal truncations. (b–d) Examples of docking analysis in ADpm and ADpm-9. b ADpm, c ADpm-9, d Superimposed structures. Note, residues shown in red indicate the interact amino acids keep consistent in the truncated and wild-type ones. Figure S5. Effect of C-terminal His-tag on GFP degradation. C-terminal 6His-tag was added to GFP with conserved degron. Figure S6. Effects of AD degron elimination on fermentation. Headspace analysis of ADse (Red) and ADse-10 (Blue). U1–U7, unidentified peaks. Figure S7. Effects of AD degron elimination on cell growth. (a–d) Growth curves of ADse (black) and ADse-10 (red) in M9 medium containing different concentrations (0.5, 1, 2 and 10 g/L) of glucose. Table S1. Statistical analysis of amino acid usage frequency (%) at C-terminus in 371 bacterial ADs. [file 13068_2020_1753_MOESM1_ESM.docx]

**Additional file 1**

**
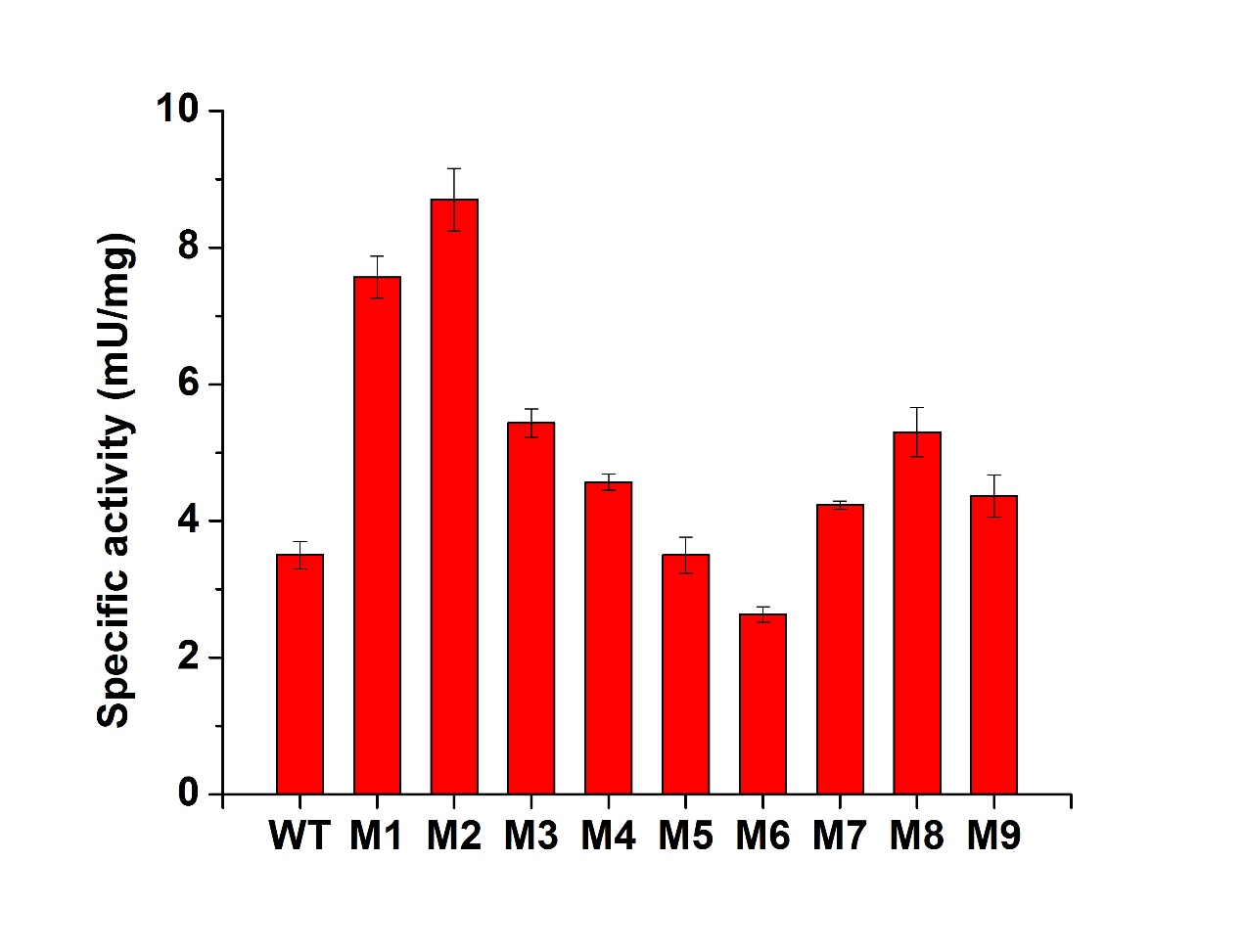
**

**Additional file 1: Figure S1.** Whole cell lysis activity analysis of different mutants. M2 give the highest activity, which has a premature stop codon in the C-terminal region. Note, WT, wild type ADpm, M1-M9 different mutants.


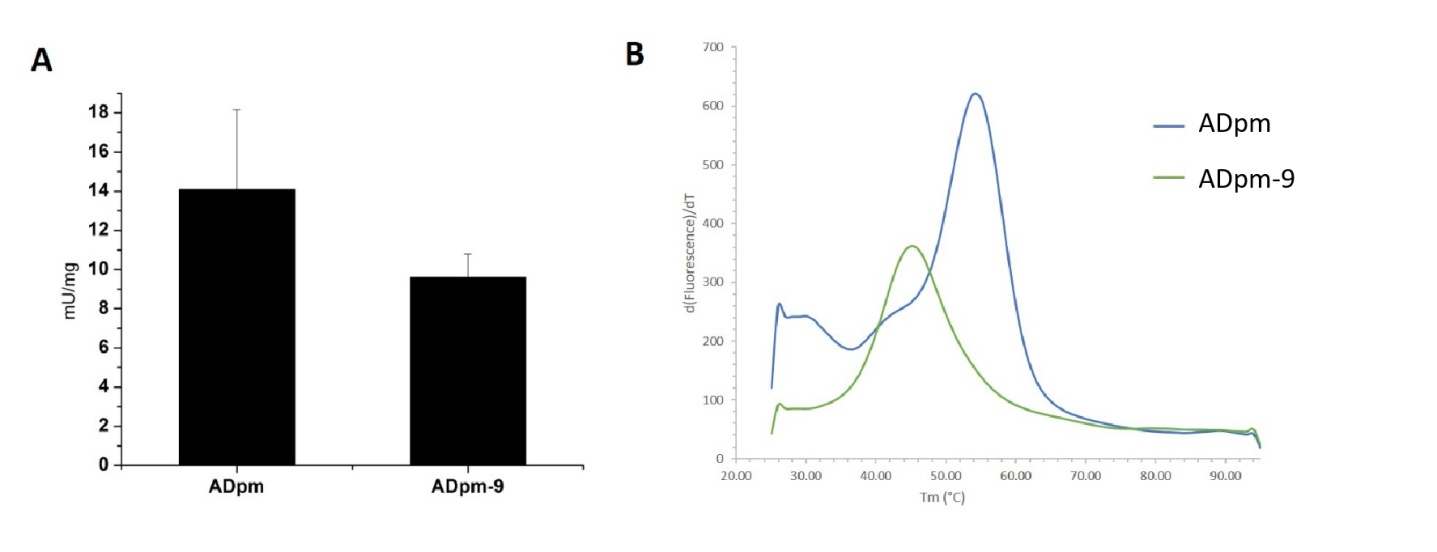


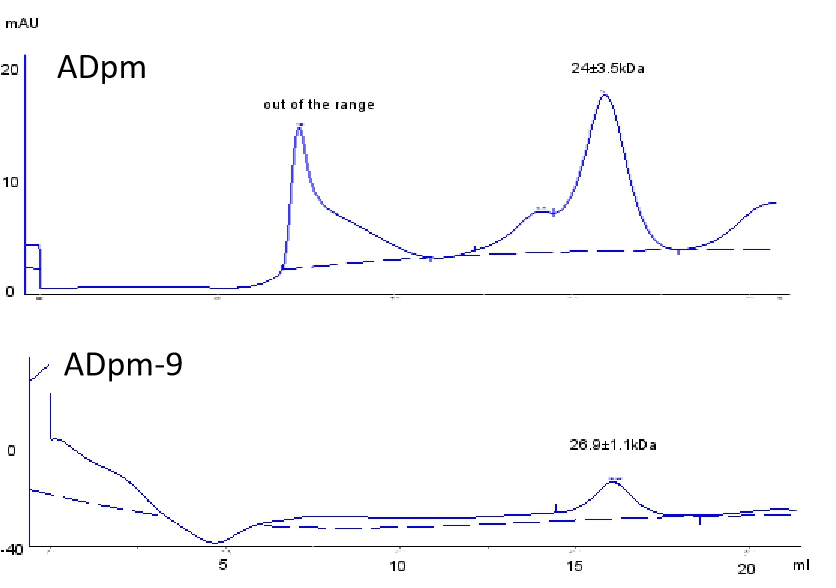


**C**

**Additional file 1: Figure S2.** Comparison of ADpm and ADpm-9. (A) Specific activity of ADpm and ADpm-9. (B) Relative protein thermal stability estimated using SYPRO Orange dye. (C) Investigation of oligomeric state of ADpm and ADpm-9 using size exclusion chromatography.


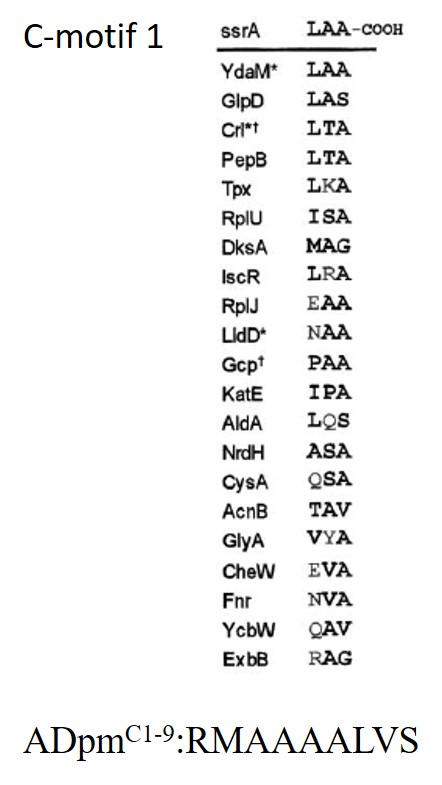


**Additional file 1: Figure S3.** Comparison of C-terminal of ADpm and C-motif 1 [35] of protease trapped proteins


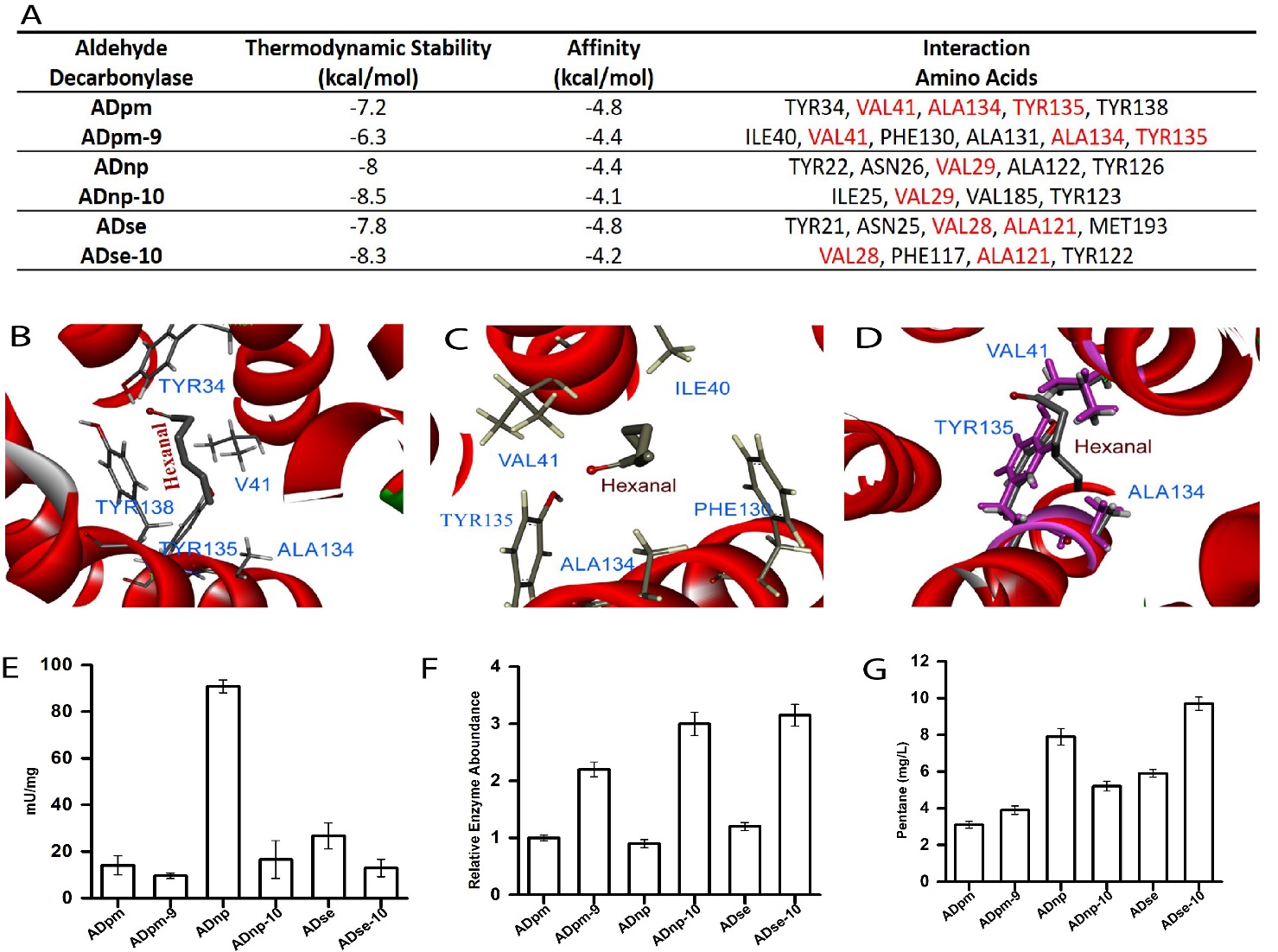


**Additional file 1: Figure S4.** Modelling results of degron subtraction in three representative ADs from *P. marinus* (ADpm), *N. punctiforme* (ADnp) and *S. elongates* (ADse). (A) Thermostability and hexanal docking analysis of three ADs and their C-terminal truncations. (B-D) Examples of docking analysis in ADpm and ADpm-9. (B) ADpm, (C) ADpm-9, (D) Superimposed structures. Note, residues shown in red indicate the interact amino acids keep consistent in the truncated and wild-type ones.


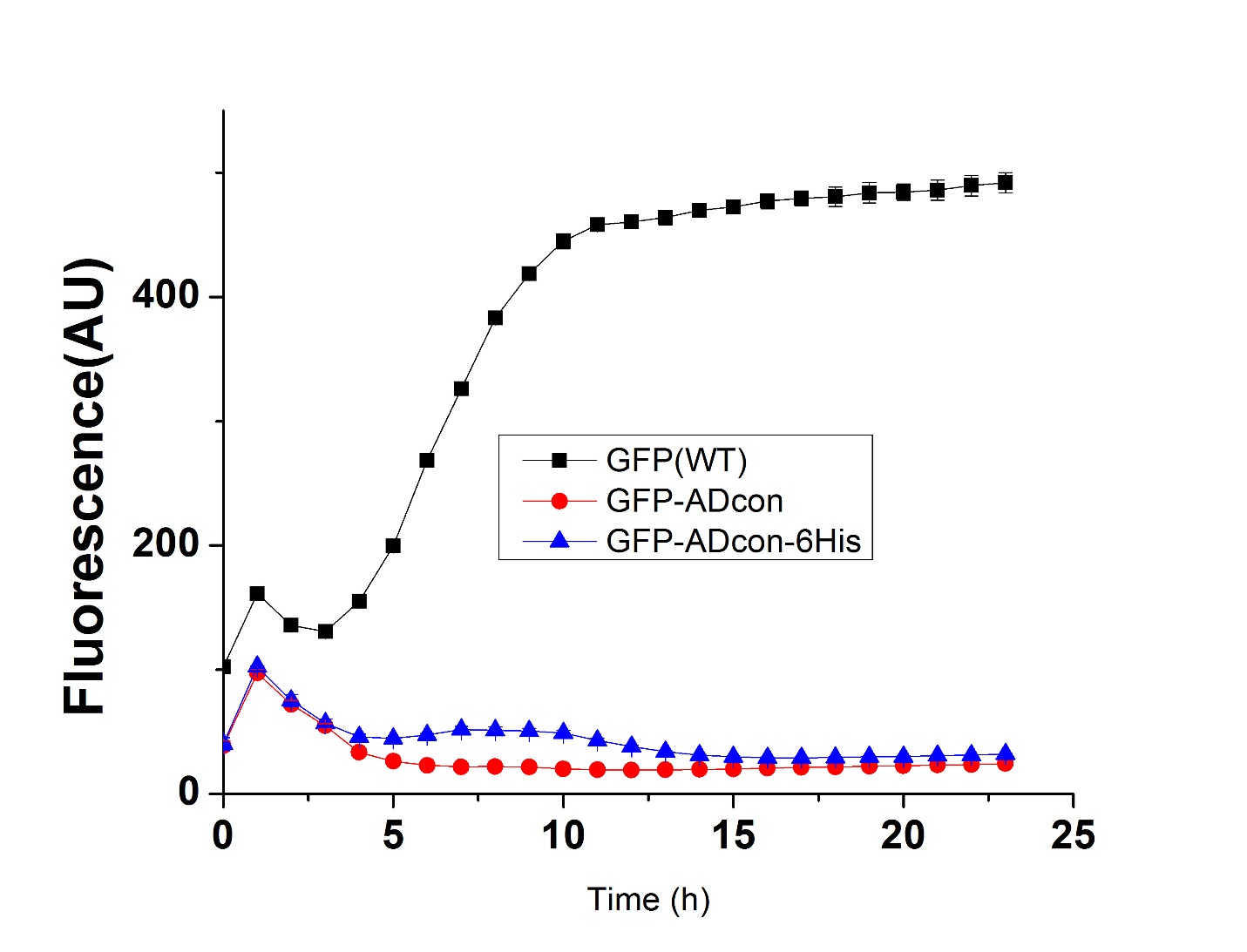


**Additional file 1: Figure S5.** Effect of C-terminal His-tag on GFP degradation. C-terminal 6His-tag was added to GFP with conserved degron.


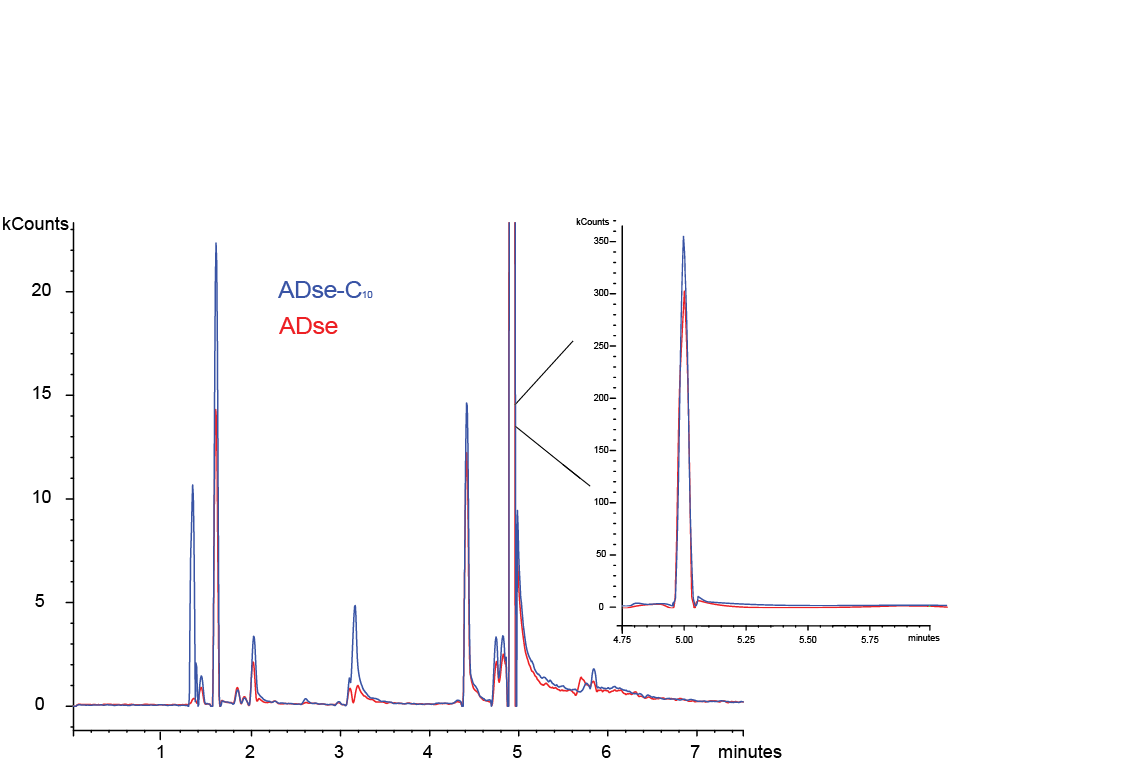


U7

U2

U3

U4

U56

U1

Pentanee

**Additional file 1: Figure S6.** Effects of AD degron elimination on fermentation. Headspace analysis of ADse (Red) and ADse-10 (Blue). Note, U1-U7, unidentified peaks.


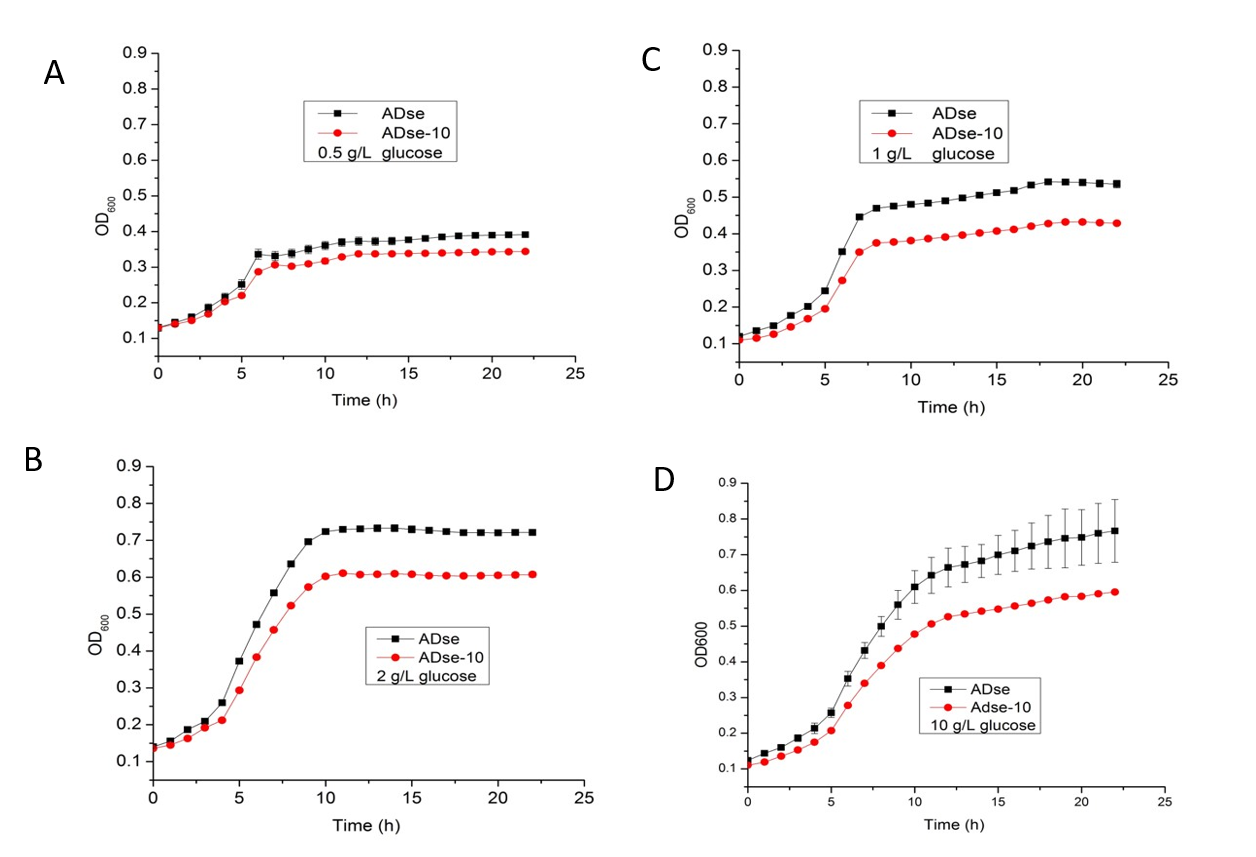


**Additional file 1: Figure S7.** Effects of AD degron elimination on cell growth. (A-D) Growth curves of ADse (black) and ADse-10 (red) in M9 medium containing different concentrations (0.5, 1, 2 and 10 g/L) of glucose.

**Additional file1: Table S1.** Statistical analysis of amino acid usage frequency (%) at C-terminus in 371 bacterial ADs.

|  | C10 | C9 | C8 | C7 | C6 | C5 | C4 | C3 | C2 | C1 |
| --- | --- | --- | --- | --- | --- | --- | --- | --- | --- | --- |
| A | 16.17 | 8.89 | 4.31 | 67.92 | 32.61 | 25.61 | 31.27 | 27.49 | 54.18 | 45.55 |
| R | 49.60 | 19.95 | 9.43 | 2.16 | 1.62 | 0.27 | 2.16 | 8.89 | 1.08 | 1.08 |
| N | 0.27 | 0.00 | 0.27 | 1.35 | 0.27 | 0.00 | 0.27 | 0.27 | 1.35 | 0.27 |
| D | 0.54 | 1.08 | 0.54 | 0.27 | 0.00 | 0.27 | 0.27 | 0.81 | 0.27 | 0.00 |
| C | 0.00 | 0.00 | 0.00 | 0.00 | 0.00 | 0.00 | 0.00 | 0.27 | 1.35 | 0.27 |
| E | 1.62 | 0.54 | 0.27 | 0.00 | 0.27 | 0.54 | 0.81 | 0.54 | 1.08 | 0.54 |
| Q | 0.27 | 0.27 | 1.35 | 1.35 | 3.50 | 2.96 | 1.08 | 0.81 | 0.81 | 0.27 |
| G | 0.27 | 0.27 | 2.16 | 1.62 | 2.43 | 56.06 | 3.23 | 2.96 | 6.20 | 7.55 |
| H | 0.00 | 0.81 | 0.00 | 0.54 | 4.85 | 0.27 | 0.27 | 0.00 | 0.27 | 0.54 |
| I | 9.16 | 2.70 | 0.81 | 0.27 | 1.35 | 0.27 | 1.08 | 3.77 | 6.47 | 5.93 |
| L | 3.50 | 27.76 | 2.43 | 2.16 | 2.16 | 4.85 | 56.33 | 22.64 | 3.23 | 1.08 |
| K | 5.39 | 2.70 | 0.54 | 0.00 | 0.00 | 0.54 | 0.27 | 2.70 | 0.81 | 0.00 |
| M | 4.04 | 28.84 | 21.29 | 9.16 | 4.04 | 5.12 | 0.00 | 0.54 | 1.08 | 0.00 |
| F | 0.27 | 0.00 | 0.00 | 0.54 | 0.00 | 0.00 | 0.00 | 0.00 | 0.27 | 0.27 |
| P | 1.35 | 0.00 | 0.00 | 1.08 | 0.27 | 0.27 | 0.00 | 0.81 | 0.81 | 1.35 |
| S | 0.81 | 2.96 | 55.26 | 8.36 | 2.43 | 1.08 | 1.08 | 3.77 | 3.50 | 6.20 |
| T | 4.85 | 1.62 | 1.35 | 0.27 | 0.81 | 0.27 | 0.81 | 16.98 | 7.82 | 0.81 |
| W | 0.00 | 0.00 | 0.00 | 0.00 | 0.00 | 0.00 | 0.00 | 0.00 | 0.00 | 0.00 |
| Y | 0.00 | 0.54 | 0.00 | 0.81 | 42.86 | 1.08 | 0.00 | 0.27 | 0.27 | 0.00 |
| V | 1.89 | 1.08 | 0.00 | 2.16 | 0.54 | 0.54 | 1.08 | 6.47 | 9.16 | 28.30 |
